# Reinventing the wheal: A review of online misinformation and conspiracy theories in urticaria

**DOI:** 10.1111/cea.14246

**Published:** 2022-10-20

**Authors:** Paula Finnegan, Michelle Murphy, Cathal O'Connor

**Affiliations:** ^1^ Dermatology South Infirmary Victoria University Hospital Cork Ireland; ^2^ Medicine University College Cork Cork Ireland

**Key Messages**

- Chronic urticaria is complex, distressing and can be recalcitrant, leaving patients susceptible to health misinformation.
- Key areas of misinformation included causes, treatments, diet and supplementation and alternative treatments.
- The allergy and dermatology communities should be vigilant regarding urticaria‐related misinformation.

To the editor,

‘Aodh tochais agus díth igne ort!’ (seanmhallacht) – may you be covered in hives without nails to scratch them! (ancient Irish curse).

Urticaria involves pruritic, transient, but recurrent wheals and/or angio‐oedema that can occur spontaneously or be inducible.^1^ Chronic urticaria (CU) refers to frequent wheals lasting 6 weeks or longer. CU spontaneously resolves within 5 years in 50% of patients, but 20% have ongoing disease after 10 years and 10% are affected after 20 years.^2^ Most (80%) patients with CU respond to standard or high‐dose antihistamines, and two‐thirds of antihistamine‐resistant CU responds to omalizumab.^1^ Despite these effective treatments, CU is an extremely distressing condition and can have a significant impact on patient quality of life due to recurrent symptoms and unpredictable course.^3^ One third of the patients with CU have depression, anxiety, sleep disorders or impaired school or work performance.^2^

Health misinformation can be defined as a health‐related claim that is not consistent with scientific consensus and is not biologically plausible. Patients with CU increasingly use electronic communication in the form of social media, computers and smart devices for unfiltered information, putting them at risk of misinformation.^4^ We aimed to examine the content of misinformation and conspiracy theories available online related to urticaria.

A PubMed literature search was performed, using the terms ‘urticaria’ AND ‘misinformation’ OR ‘disinformation’ OR ‘conspiracy theory. This yielded 1220 results, which were reviewed for suitability by authors PF and COC, with one paper deemed appropriate for inclusion as it included content of misinformation.^3^

An informal Google search was also carried out using combinations of the terms ‘urticaria’, ‘hives’ and ‘misinformation’, ‘disinformation’ and ‘conspiracy theory’. Information was collected from the first 10 pages of each Google search. Further targeted searches were also conducted on social media including Twitter, Facebook, Instagram and TikTok.

Key areas that were identified in the search included allergy being proposed as a ‘cause’ of CU, suggestions that there was no effective or safe medical treatment for CU, exclusion diets or ‘natural’ dietary or nutritional supplements and alternative treatments which were usually promoted by individuals or business selling the recommended products (Figure 1).

**FIGURE 1:**
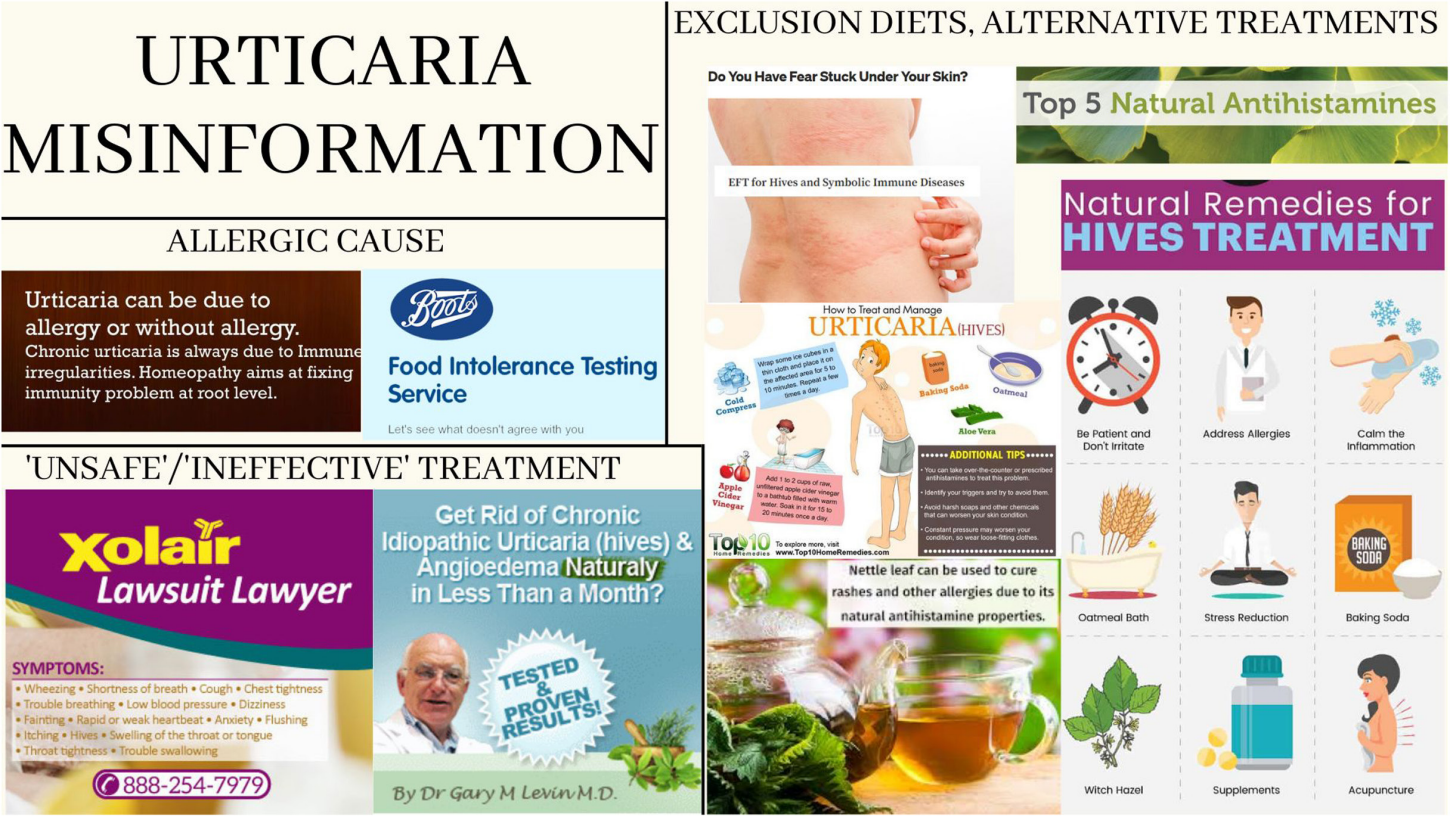
Misinformation in urticaria—images from various websites.

Allergies were frequently mentioned as a cause of CU. While acute urticaria immediately following exposure to a food allergen or aeroallergen is consistent with IgE‐mediated allergy, CU lasting more than 6 weeks is not consistent with allergy, and no allergy tests are recommended in the assessment of CU.^5^ Other ‘hidden’ allergens were frequently mentioned. Expensive testing was often advertised to assess for allergies, mostly based on IgG levels, which are inappropriate for diagnosing food allergy, with higher levels usually representing tolerance rather than allergy.^6^ Other posts suggested that all patients with CU should have skin prick testing or patch testing, often failing to demonstrate an awareness of the difference between the two modalities.

Many social media accounts propagated the myth that there was no effective or safe medical treatment for CU. While some patients may have suboptimal responses, there are many effective and safe medical treatments for urticaria such as antihistamines, which have been commercially available since 1942.^7^ Anti‐IgE monoclonal antibody therapy such as omalizumab is available for more severe CU and is highly efficacious and well‐tolerated.^1^ Some websites labelled antihistamines as ‘toxic’ and falsely promoted the need for a prolonged anti‐histamine ‘detox’ for natural treatments to become effective. Omalizumab has also been vilified online, in particular on Facebook, where a ‘Xolair lawsuits’ group has been formed to spread misinformation about a falsely elevated risk of major adverse events.

Low histamine diets were frequently recommended for CU, avoiding mature cheeses (e.g. parmesan or blue cheese), alcohol (especially red wine), pickled or canned foods, smoked meats (e.g. salami), fish (tuna, sardines, anchovy fillets and salmon), fermented products, shellfish, beans, pulses, vinegar and foods with preservatives or artificial colouring. Other dietary regimens mentioned include a low sulphite diet, a low tyramine diet, avoiding histamine‐releasing foods (citrus fruit, tomatoes, chocolate, nuts), increasing intake of histamine‐reducing foods and avoiding food with additives or pesticides. Most recent guidelines state that dietary exclusions should not be routinely advised, and that there is insufficient evidence at present to recommend any dietary supplementation.^1^, ^2^

A myriad of alternative treatments for urticaria were proposed online. These included dietary supplements, hypnotherapy, kinesiology, acupuncture, and homeopathy. A ‘medical medium’ claimed that urticaria is caused by ‘fear trapped beneath the skin’ and offered the ‘Emotional Freedom Technique’ as treatment. Homeopathic and alternative remedies proposed online included witchhazel, baking soda, quercetin, evening primrose, vitamin B12, vitamin C, vitamin D, fish oil, apple cider vinegar, turmeric, virgin coconut oil, green tea, tea tree oil, ginger, basil, nettles (capsules or steamed), shea butter and lavender oil. A Chinese folk remedy recommended application of a balm composed of brown sugar, ginger, vinegar and warm water. Other ‘home remedies’ were frequently recommended. In Ayurvedic medicine, urticaria is referred to as *Sheetapitta*, and suggested herbal remedies included Haridra (*Curcuma longa*), Neem (*Azadirachta indica*), Shirish (*Albezzia lebbock*), Ashwagandha (*Withania somnifera*), Shirish (*A. lebbock*), Guduchi leaves, aloe vera, black pepper powder, or desi ghee. None of these agents have plausible potent antihistamine effects, and there is no evidence that any of them are beneficial in CU.

Chronic urticaria can have a major psychological impact, leaving patients vulnerable to misinformation, and desperate for a cure for their uncontrolled itch. Patients may not find their prescribed treatments useful, or they may be concerned about potential adverse effects. Their desperation, coupled with the exorbitant amount of accessible health misinformation available online, can lead to patients opting for alternative treatment options that have no evidence of benefit nor plausible mechanism of action. Recommendations to counter health misinformation include enhancing surveillance, understanding psychological drivers, recognizing consequences, focusing on vulnerable populations, and developing effective responses.^8^ Recently, a ‘social media history’ has been proposed by dermatologists to combat misinformation in clinic.^9^ Allergists and dermatologists should be aware of urticaria‐related misinformation and actively aim to counteract it with patient education and evidence‐based guidelines.

## AUTHOR CONTRIBUTION

Paula Finnegan performed the literature search, wrote the first draft of the manuscript, and reviewed updates of the manuscript. Michelle Murphy and Cathal O'Connor identified the literature gap on the topic, helped with the first draft of the manuscript, and reviewed the manuscript. Cathal O'Connor created the figure.

## CONFLICT OF INTEREST

None.

## Data Availability

The data that support the findings of this study are available from the corresponding author upon reasonable request.
